# Zone Specific Fractal Dimension of Retinal Images as Predictor of Stroke Incidence

**DOI:** 10.1155/2014/467462

**Published:** 2014-11-18

**Authors:** Behzad Aliahmad, Dinesh Kant Kumar, Hao Hao, Premith Unnikrishnan, Mohd Zulfaezal Che Azemin, Ryo Kawasaki, Paul Mitchell

**Affiliations:** ^1^School of Electrical and Computer Engineering, RMIT University, 124 Latrobe Street, Melbourne, VIC 3000, Australia; ^2^Department of Optometry and Visual Science, Kulliyyah of Allied Health Sciences (KAHS), International Islamic University Malaysia (IIUM), Bandar Indera Mahkota, 25200 Kuantan, Pahang, Malaysia; ^3^Department of Public Health, Yamagata University Faculty of Medicine, 2-2-2 Iida-Nishi, Yamagata-shi, Yamagata 990-9585, Japan; ^4^Centre for Vision Research, Department of Ophthalmology and Westmead Millennium Institute, University of Sydney, 176 Hawkesbury Road, Westmead, NSW 2145, Australia

## Abstract

Fractal dimensions (FDs) are frequently used for summarizing the complexity of retinal vascular. However, previous techniques on this topic were not zone specific. A new methodology to measure FD of a specific zone in retinal images has been developed and tested as a marker for stroke prediction. Higuchi's fractal dimension was measured in circumferential direction (FDC) with respect to optic disk (OD), in three concentric regions between OD boundary and 1.5 OD diameter from its margin. The significance of its association with future episode of stroke event was tested using the Blue Mountain Eye Study (BMES) database and compared against spectrum fractal dimension (SFD) and box-counting (BC) dimension. Kruskal-Wallis analysis revealed FDC as a better predictor of stroke (*H* = 5.80, *P* = 0.016, *α* = 0.05) compared with SFD (*H* = 0.51, *P* = 0.475, *α* = 0.05) and BC (*H* = 0.41, *P* = 0.520, *α* = 0.05) with overall lower median value for the cases compared to the control group. This work has shown that there is a significant association between zone specific FDC of eye fundus images with future episode of stroke while this difference is not significant when other FD methods are employed.

## 1. Introduction

Retinal imaging provides a window for in vivo visualization of the eye microvasculature which has physiological and embryological similarities with micro vessels elsewhere in the body [1, 2]. It is now commonly used for research applications in the area of screening ocular disease and risk assessment of nonocular diseases such as diabetes [3], hypertension [4, 5], and stroke incidence [6–8].

Eye fundus imaging provides noninvasive viewing of the retinal vasculature and has been shown to have a number of anatomic, physiological, and embryological similarities with cerebral vasculature [2, 7]. The observable changes to retinal vasculature have been associated with various cardiovascular and metabolic diseases including stroke risk assessment [2, 7] and assessment of high blood pressure, diabetes, arteriosclerosis, and other cardiovascular diseases [9]. Therefore, a number of automatic and semiautomatic retinal image analysis tools have been developed over the past 10 years [10] to improve the reliability and make the procedure more robust and cost effective.

Fractal dimension (FD) has been frequently employed in many studies for quantification of the complexity of retinal microvasculature pattern and as a potential indicator of the complexity of cerebral vasculature branching pattern. It is unitless and in general it is measured as ratio of how the geometrical details in a pattern change with respect to different magnification factors (scales). FD measures self-similarity in structural characteristics of retinal branching pattern, and its value increases with increase in the structural complexity [11]. Therefore, a number of studies have shown that FD of the retinal image can be used as disease marker [12–14], associated with relative changes in retinal vasculature and geometry. For instance, the box-counting (BC) method was employed by Doubal et al. [15] to study the prevalence of Lacunar stroke. They found a decreasing trend for FD and an association with age factor due to loss of branching complexity. However, BC is susceptible to the image segmentation and skeletonization quality, essential preprocessing steps for BC computation. To overcome the segmentation limitation, Azemin et al. [16] proposed the Fourier based spectral fractal dimension (SFD) and found association of SFD with age factor. This was later tested by Kawasaki et al. [6] in a study to determine the difference between healthy subjects and those who later suffered from stroke and reported a statistical significance (*P* = 0.044) between the case and control groups.

FD is a convenient method for summarizing the image properties and provides a global measure for the entire image. This makes it useful for automated analysis of the retinal images. However, FD is nonspecific to a region of the image and does not provide the examiner the ability to study local regions.

In this work, we have developed a method to measure FD of specific concentric zones around optic disc (OD). The technique does not require image segmentation or vessel boundary detection preprocessing stage. After automatically detecting the OD, the image is scanned in circles and then the Higuchi's one-dimensional (1D) FD is estimated for the region of interest. A comparison was made with the current state of the art FD techniques, SFD [6] and BC [13], in terms of association between control participants and the people who later developed an episode of stroke in a case-control study nested in a population-based cohort study of the Blue Mountains Eye.

## 2. Materials

### 2.1. Dataset

The retinal images from the Blue Mountains Eye Study (BMES), a population-based study conducted in a suburban region west of Sydney, Australia, were analysed [17, 18]. The participant's age range was 50–89 years. Images of the retina from both eyes of the study participants were obtained using a Zeiss FF3 fundus camera having 30-degree field. The photographs were taken after pupil dilation. The images were digitized using a Cannon FS2710 scanner with maximum resolution of 2720 dpi in 24-bit colour format. Among the total number of 1532 optic disk (OD) centred images (3888 × 2592 pixels) in the database, total number of 104 subjects were confirmed with an episode of stroke after five-year follow-up, comprising of 21 stroke events, 86 stroke-related deaths, and 3 persons overlapping with both. Self-reported stroke events were validated against medical records of physician diagnosis based on the World Health Organization Monitoring Trends and Determinants in Cardiovascular Disease (WHO-MONICA) plus evidence from computed tomography or magnetic resonance imaging [19].

### 2.2. Data Management

Images from the left eyes were analysed as there was a high correlation between left and right eye images (*r* = 0.63) reported by Kawasaki et al. [6] and they had only used left eye images in their study in order to maintain independence of the samples. Out of 104 available stroke cases, 3 images were ungradable due to quality and contrast problem. Among the rest, 52 cases were diagnosed with hypertension and 6 with diabetes which were discarded from the analysis, leaving a total number of 46 stroke cases. An independent third party ophthalmologist confirmed matching of the 46 cases with 39 controls prior to analysis based on the age (mean (SD) = 67.76 (5.72)), metric body mass index (BMI (Kg/m^2^)) (26.32 (4.35)), blood pressure (mmHg) (systolic: 150.64 (18.94), diastolic 83.40 (10.35)), and history of smoking. The matching control set was of people who did not have history of any systemic disease including stroke, hypertension, and diabetes at the baseline and did not develop any of them during the period of the study.  Table 1 compares the characteristics of stoke cases and matched controls.

## 3. Retinal Image Analysis

**Figure 1:**
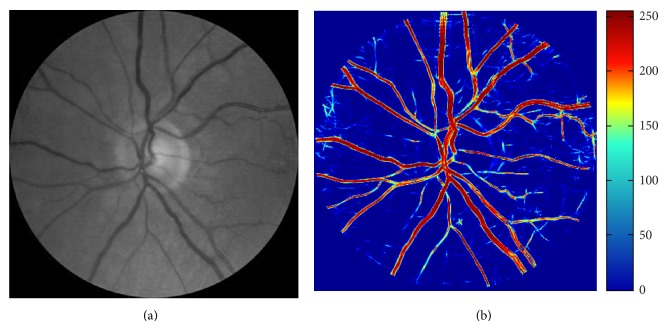
Retinal image enhancement. (a) Retinal image (green channel), (b) enhanced retinal image and color representation of the gray scale intensity values.

The inverted green channel was used [20] as this provided better vessel to background contrast compared to the other two channels (red and blue). The images were cropped using a mask of size 1960 × 1960 pixels to cover a region of interest (ROI) corresponding to a circle of 4 OD diameter (*D*_OD_) centered at OD center. The OD center and boundary were identified manually by the grader [16]. The cropped image was then downsampled to 400 × 400 pixels (Figure 1(a)) to reduce the computational complexity similar to other studies [16, 20].

### 3.1. Vessel Enhancement

Image enhancement of the retinal images was performed to compensate for a set of degrading artifacts. The artifacts may be present due to uneven and improper illumination inside the eye, eye movement, and ocular media opacity which will have impairing effect on the analysis outcome [16]. Figure 1(a) shows an example of a low contrast retinal image with poor illumination. Based on the works by Soares et al. [21], 2D Gabor wavelet was employed as a directional matched filter for retinal image enhancement to extract the vascular network over 18 equally spaced orientations (10° steps from 0 to 170°) and 5 different scales (4, 5, 6, 7, and 8). The wavelet parameters including the elongation factor and the horizontal and vertical frequencies were set to 4 and {0, 3}, respectively, to match the previous works [16, 21]. After this, the intensity of the transformed images was normalized and the class-conditional probability density function was computed to obtain gray scale posteriori vessel images [21]. The output of the probability density function was a set of values in the range of 0 to 1 associated with the likelihood of a pixel belonging to background or vessel with 1 being considered as vessel. However, for analysis and color representation of posterior data the values were transformed into a new integer range of 0 to 255. An example of an enhanced retinal image (posteriori) with color representation of the gray scale intensity values has been shown in Figure 1(b).

### 3.2. Higuchi's Fractal Dimension

**Figure 2:**
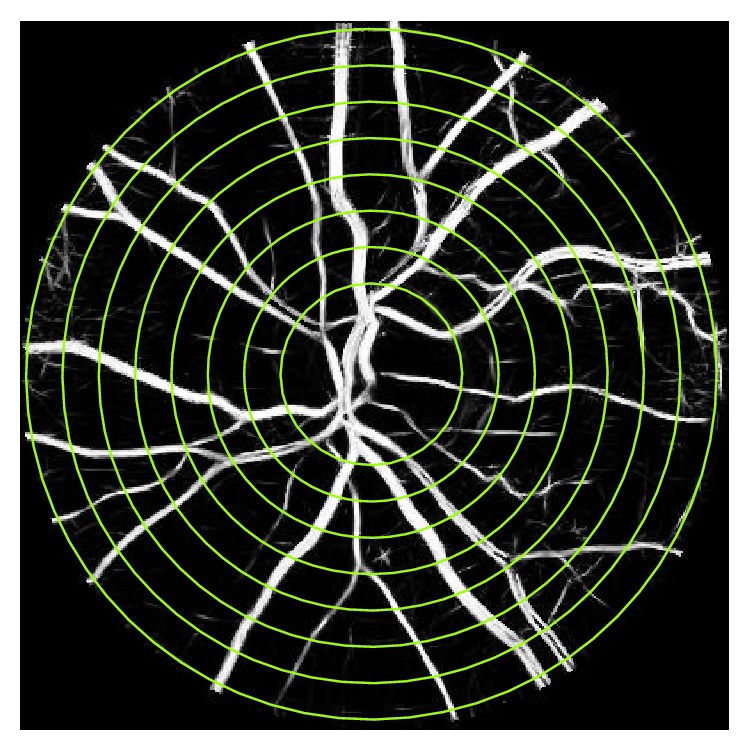
Demonstration of circular scanning paths using an enhanced retinal image. The scanning paths have been demonstrated with several pixels intervals for better visualization purpose.

Higuchi's technique measures the FD of a set of points in the form of 1D series [22]. To estimate the Higuchi's FD of an image, the image intensities have to be scanned, which may be along a set of directions, such as horizontal, vertical, diagonal, or circular [23]. Selection of an appropriate scanning method depends on image features of interest. In this study concentric circles around the OD were considered for the first time in retinal image analysis. Such a scanning path intersects with most major vessels' along their cross-section (Figure 2) because the major vessels of the retina are in radial direction with respect to the OD. The circles were spaced with one-pixel intervals in the ROI with the innermost circle having its radius one pixel greater than that of the OD and the outermost circle with diameter 4×  *D*_OD_. Some examples of these scanning circles are shown in Figure 2.

Higuchi's FD was estimated for each of the circular scans and referred to as FD circular or FDC. The average of FDC of all circles in a zone was assigned to FDC of that zone. For detailed mathematical information regarding Higuchi's method refer to the study by Higuchi, 1988 [22].

### 3.3. Region Based Analysis of the FDC

**Figure 3:**
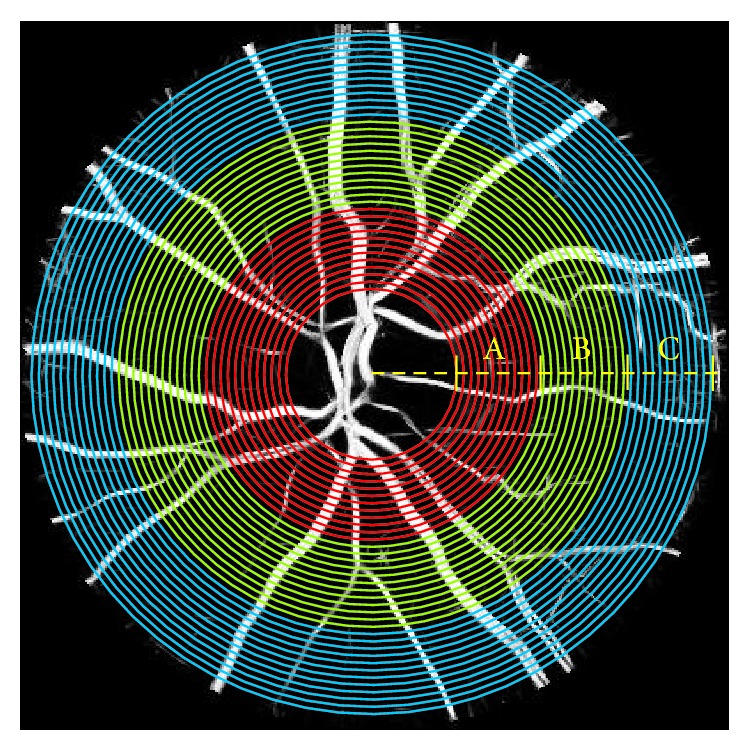
Representation of three major zones, A (red), B (green), and C (blue) around the OD with respect to one to three OD radius from OD boundary and circular scanning in the defined regions.

A zone based analysis was performed to study the potential impact of the selection of the zone and to identify the zone most indicative of the risk of disease (stroke). A mask of concentric circles (circular scanning paths) centered at OD center was created for each image, with the smallest circle corresponding to the OD boundary with diameter *D*_OD_. The ROI was divided into three concentric zones (A, B, and C), with zone A corresponding to a circumferential region between 0 and 0.5 *D*_OD_ from the OD margin. Similarly, zones B and C were defined between 0.5 to 1 and 1 to 1.5 *D*_OD_ from its margin, respectively (Figure 3). The 1D data series were separately obtained for each zone on the circular paths and the average of all FD values in each zone was calculated. All seven possible combinations of the zones, A, B, C, AB, BC, AC, and ABC, were considered and associations with case/control groups for FDC_A_, FDC_B_, FDC_C_, FDC_AB_, FDC_BC_, FDC_AC_, and FDC_ABC_ were tested.

### 3.4. Other FD Measurement Techniques

FDC was compared with spectrum fractal dimension (SFD) [6] and box-counting (BC) technique [13].

SFD, also known as Fourier fractal dimension (FFD), has been recently employed for retinal image analysis in the area of stroke risk assessment [6, 20] and age analysis [16]. SFD was obtained by finding magnitude of the Fourier transform of vessel-enhanced retinal images (grayscale image) and plotting it versus the frequency. The slope of the plot on a logarithmic scale was considered as the spectrum (Fourier) fractal dimension.

Except SFD, BC has also been generally accepted and employed for quantifying complexity of retinal vasculature [3, 13] and disease analyses [15]. This method is applicable for binary images and involves superimposing the structure (vascular network in retinal images) with a grid of boxes with varying (either deceasing or increasing) side length of sizes and counting the number of boxes corresponding to each size, containing at least one pixel of the structure. The slope of the best fitting line to the data points on a log-log plot of number of boxes versus side length size corresponds to the box-counting fractal dimension.

## 4. Statistical Analysis

Two different sets of tests were performed. The first test compared FDC with other FD techniques (i.e., BC and SFD). The second test used FDC and identified which of the zones were most relevant for distinguishing between case and control. For the zone based analysis, the associations of FDC in all seven possible regions with case and control groups were compared.

Statistical analyses were performed using Minitab 16 (Minitab Inc.). For each FD measurement technique, the measured values between the subjects were compared and the significance of association (*P* values) between case and control groups was tested using the nonparametric Kruskal-Wallis analysis as the condition of normality (i.e., normal distribution of data) was not satisfied. The Kruskal-Wallis is one way analysis of variance by ranks and is a nonparametric statistical method to test whether the population medians (*η*) on a dependent variable are the same across all levels of a factor, while assuming the null hypothesis of H_0_: *η*_Case_ = *η*_Control_ versus the alternative H_1_: *η*_Case_ ≠ *η*_Control_. The ranking is performed by arranging and labeling the population data from low to high starting from 1. This test is suitable for the populations with two levels of independent variables.

For the zone based analysis, all the seven FDCs corresponding to the regions around OD were compared accordingly using Kruskal-Wallis analysis. The 95% confidence interval (CI) for the difference in population medians (*η*_Case_ − *η*_Control_) was also obtained by nonparametric pairwise comparison between the case and control groups using the Mann-Whitney as a follow-up measure. The Mann-Whitney test, which is also known as Wilcoxon rank-sum test, is an alternative to two-sample *t*-test for measuring the statistical significance between two populations when the data are not normally distributed.

## 5. Results

Table 2 shows the result from statistical significance test between FDs of the case and control groups according to the method by which the FD was calculated. The results show that the *η*_Case_  (1.981) was lower for FDC compared to the control group (*η*_Case_ = 1.987) while this was opposite for BC (*η*_Case_ = 1.653 > *η*_Control_ = 1.648). No difference between the medians of case and control was observed for SFD (*η*_Case_ = 1.335 and *η*_Control_ = 1.335). From columns 8, 9, and 10, it is observed that there is a statistically significant difference (*H* = 5.80, *P* = 0.016, *α* = 0.05) between FDC of the case and control groups with 95% CI *η*_Case_ − *η*_Control_ = −0.0087, −0.0007. However, no statistical significance was detected for other FD techniques (all *P* values > 0.05).

The comparison of the FDC for different zones of the retinal images is shown in Table 3. The first column lists the zones where the FDC was calculated. From this table, it is observed that the two groups (case and controls) are not statistically significant based upon the FDC_A_ alone (*H* = 2.60, *P* = 0.107, *α* = 0.05, 95% CI (−0.0073, 0.0009)). The results are similar for regions C (FDC_C_) and FDCs of regions A and C together (FDC_AC⁡_). However, FDC_B_, FDC_AB_, FDC_BC_, and FDC_ABC_ (FDC from Table 2) are found to be statistically significant with the *P* values of 0.016, 0.025, 0.031, and 0.016 (*α* = 0.05), respectively.

## 6. Discussions

FD measurement techniques are convenient methods for summarizing the retinal vessel complexity and have been found to be useful in case/control studies [3, 6, 24]. Unlike vessel caliber measurement techniques, FD measurement in general does not require manual supervision and summarizes the entire image into a single value. However, while the vessel caliber summarizing techniques are specific to zones within the retinal images [8], the FD techniques do not allow the examiner to target any specific zone in the retinal image but estimate the FD of the entire image.

We have introduced a new method to obtain Higuchi's fractal dimension of retinal vasculature. In this method, the image is scanned using circles around the optic disc (OD) and is referred to as circular FD (FDC). This method allows the scanning within a specific zone of OD centric retinal image. The other advantage of this method is that unlike box-counting FD, this does not require image segmentation [25] and can be directly applied to gray scale images as image segmentation may cause loss of vessel caliber information [26] and require manual corrections for possible vessel discontinuities and background noise added during the process. Therefore, segmentation can be a potential source of error in FD measurement.

In order to test its efficacy in distinguishing between cases and controls, it was compared with BC [13] and SFD [6] methods when applied to the same subsample of BMES population for the stroke cases, while excluding the hypertensive and diabetic cases. The results show that FDC had a significant relationship with the five-year stroke event and mortality (*P* = 0.016, *α* = 0.05) while other fractal measures did not show any significant association for these subsamples (all *P* values > 0.05). This could be due to our new exclusion criteria and limiting the dataset only to stroke factor (i.e., removing the hypertensive and diabetic stroke cases from the analysis as two confounding factors). The median FDC for the entire image was lower (1.981) for the cases compared to the controls (1.987). This indicates that reduction in the complexity of the retinal vasculature is an indicator of disease and is comparable with findings of Lipsitz and Goldberger [27] which is associated with functional loss [28]. Such loss of complexity has also been observed associated with ageing and disease in cardiac activity [29], neural system [30], electromyogram (EMG) [31–33], and general physiological measures [28]. This study indicates that prior to an episode of stroke, the rarefication of the retinal vasculature is associated with reduction in the fractal dimension of the eye fundus image [24].

Our experiments have confirmed that the difference between control and cases (people who later suffered from stroke) is most significant in zone B (*P* = 0.016) which is in agreement with effectiveness of the summary vessel diameter (CRAE and CRVE) being performed only in zone B [8]. Such zone analysis is only feasible using FDC because other techniques estimate the FD of the entire image and do not have any provision to perform zone specific estimation.

Compared to other FD techniques, FDC_ABC_ (FDC) shares similar region of interest (ROI) with SFD and BC except in the optic disk (OD) area. This can be counted as another advantage of this technique as it excludes the OD region from the analysis which may have confounding effect on the analysis outcome. Therefore, the confounding effect of OD could be another reason for not seeing any significant relationship between other fractal methods and stroke factor in our analysis.

The major implication of this study is that by using FDC, it is possible to assess the risk of stroke. This research has also reconfirmed that zone B is the most relevant when attempting to observe changes in the retinal vasculature that may indicate risk of stroke. This study had a high statistical power of 88% resulting into reduced risk of type II errors (false negative).

The weakness of this study is that while FD is a convenient method in terms of summarizing the complexity of entire retinal vasculature into a single value, it does not provide information according to each vessel type (i.e., arteries and veins) which is of clinical importance. Also in this study, only the left eye images of people above the age of 49 have been considered as BMES represents narrow demographics.

## Figures and Tables

**Table 1:** Comparison of characteristics of stroke cases and matched controls.

Matched characteristics	Case (*n* = 46)	Control (*n* = 39)	*P* value
	Mean ± SD	Mean ± SD	
Age (years) ± SD	67.63 ± 5.78	67.92 ± 5.58	0.657
Systolic blood pressure (mmHg)	147.71 ± 17.70	154.10 ± 19.52	0.229
Diastolic blood pressure (mmHg)	83.69 ± 9.19	83.05 ± 11.44	0.615
Body mass index (Kg/m^2^)	26.08 ± 5.01	26.61 ± 3.51	0.868

	Ratio (%)	Ratio (%)	

Gender (female/male)	21/25 (84%)	18/21 (85%)	0.811

**Table 2:** Nonparametric Kruskal-Wallis and Mann-Whitney significance test between control and case groups on retinal images.

Method	Control (*n* = 39)			Case (*n* = 46)			95% CI^**^ for *η*_Case_ − *η*_Control_	*H* ^†^	*P* value^*^
	Median (*η*)	Average rank	*Z* ^†^	Median (*η*)	Average rank	*Z* ^†^			*η*_Case_ = *η*_Control_ versus (*η*_Case_ ≠ *η*_Control_)
FDC	1.987	50.0	2.41	1.981	37.1	−2.41	−0.0087, −0.0007	5.80	**0.016**
SFD [6]	1.335	46.1	1.07	1.335	40.4	−1.07	−0.0006, 0.0002	1.14	0.286
BC [13]	1.648	41.3	−0.58	1.653	44.4	0.58	−0.0080, 0.0140	0.33	0.563

^*^ *α* = 0.05; ^†^test statistic from Kruskal-Wallis analysis; ^**^95% CI from the Mann-Whitney test.

**Table 3:** Kruskal-Wallis and Mann-Whitney zone based FDC significance test.

	Zone based analysis								
Method	Control (*n* = 39)			Case (*n* = 46)			95% CI^**^ for *η*_Case_ − *η*_Control_	*H* ^†^	*P* value^*^ *η*_Case_ = *η*_Control_ versus (*η*_Case_ ≠ *η*_Control_)
	Median (*η*)	Average rank	*Z* ^†^	Median (*η*)	Average rank	*Z* ^†^			
FDC_A_	1.976	47.7	1.61	1.973	39.0	−1.61	−0.0073, 0.0009	2.60	0.107
FDC_B_	1.994	50.0	2.40	1.989	37.1	−2.40	−0.0079, −0.0008	5.75	0.016
FDC_C_	1.994	48.4	1.85	1.988	38.4	−1.85	−0.0118, 0.0003	3.43	0.064
FDC_AB_	1.983	49.5	2.25	1.981	37.5	−2.25	−0.0074, −0.0005	5.06	0.025
FDC_AC_	1.984	48.6	1.91	1.981	38.3	−1.91	−0.0090, 0.0000	3.66	0.056
FDC_BC_	1.993	49.3	2.15	1.988	37.7	−2.15	−0.0101, −0.0006	4.63	0.031

^*^ *α* = 0.05; ^†^test statistic from Kruskal-Wallis analysis; ^**^95% CI from the Mann-Whitney test.
